# P-27. Methicillin Resistance and Its Effect on Clinical Outcomes in Staphylococcus aureus Bacteremia-Associated Sepsis: A Retrospective Cohort Analysis

**DOI:** 10.1093/ofid/ofaf695.256

**Published:** 2026-01-11

**Authors:** Isaac Santiago Arango-Gil, Susana Montoya-Jaramillo, Isabel González-Tapias, Alejandro ardila Gil, David Alejandro Sandoval Parra, Ana Sofía Urrego-Ramírez, Manuela Cardona Gómez, Juan Jose Vesga Naranjo, Fabian Jaimes

**Affiliations:** Universidad de Antioquía, MEDELLIN, Antioquia, Colombia; Universidad de Antioquía, MEDELLIN, Antioquia, Colombia; Universidad de Antioquía, MEDELLIN, Antioquia, Colombia; Universidad de Antioquía, MEDELLIN, Antioquia, Colombia; Universidad de Antioquía, MEDELLIN, Antioquia, Colombia; Universidad de Antioquía, MEDELLIN, Antioquia, Colombia; Universidad de Antioquía, MEDELLIN, Antioquia, Colombia; Universidad de Antioquía, MEDELLIN, Antioquia, Colombia; Universidad de Antioquía, MEDELLIN, Antioquia, Colombia

## Abstract

**Background:**

*Staphylococcus aureus* is a gram-positive pathogen associated with illnesses ranging from mild to life-threatening. Over 90% of patients with *S. aureus* bacteremia (SAB) develop sepsis, and is linked to poor outcomes. Methicillin resistance may contribute to a dysregulated host response. This study aimed to assess the impact of methicillin resistance on in-hospital mortality, length of stay, and ICU admission in patients with SAB and sepsis.

**Figure:**
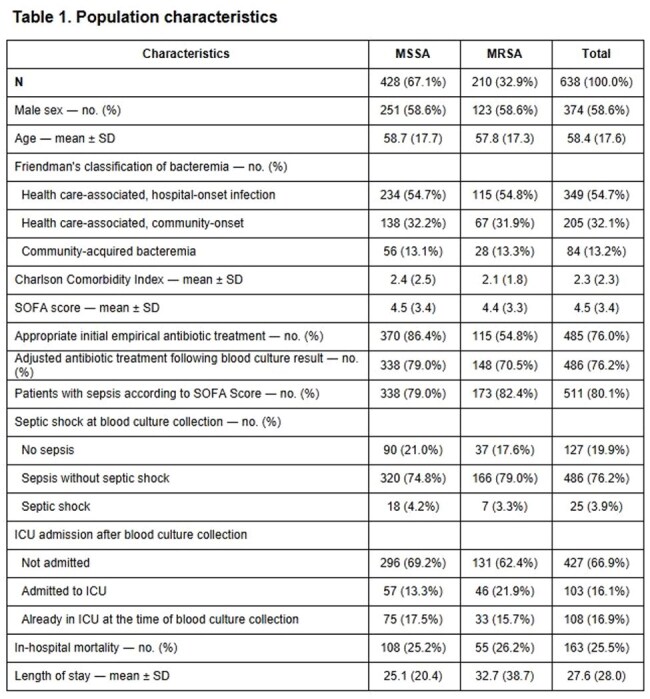
Abbreviations: MRSA: Methicillin resistant Staphylococcus aureus, MSSA, Methicillin-Susceptible Staphylococcus aureus, SOFA: Sequential Organ Failure Assessment score, ICU: Intensive care unit

**Figure:**
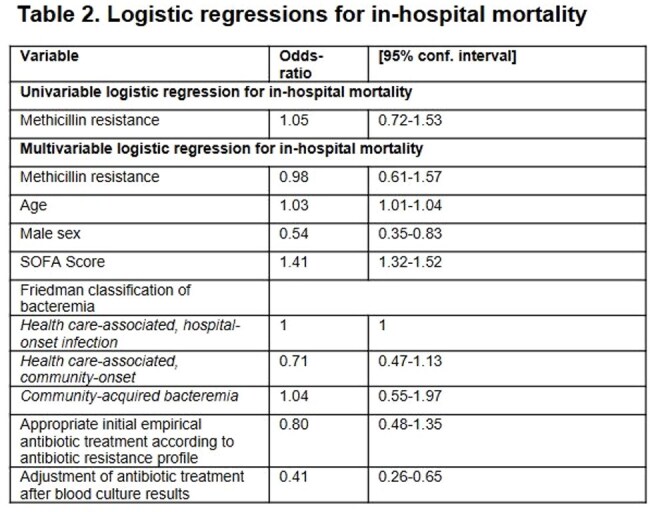
Statistical analysis: Logistic regression model adjusted for potential confounding variables. Unadjusted data present the univariable association between the exposure of interest and in-hospital mortality. Abbreviations: OR: Odds-ratio, SOFA: Sequential Organ Failure Assessment score, ICU: Intensive care unit

**Methods:**

We conducted a retrospective cohort study at a tertiary hospital in Medellín, Colombia, using data collected from 2017 to 2023. We included patients with a positive blood culture for *S. aureus*, confirmed susceptibility profiles, and sepsis or septic shock per Sepsis-3 criteria. To assess the association between methicillin resistance and outcomes, we used two regression models adjusted for potential confounders: a logistic regression model for in-hospital mortality and ICU admission, and a Cox proportional hazards model (with death as a competing risk) for hospital length of stay.

**Figure:**
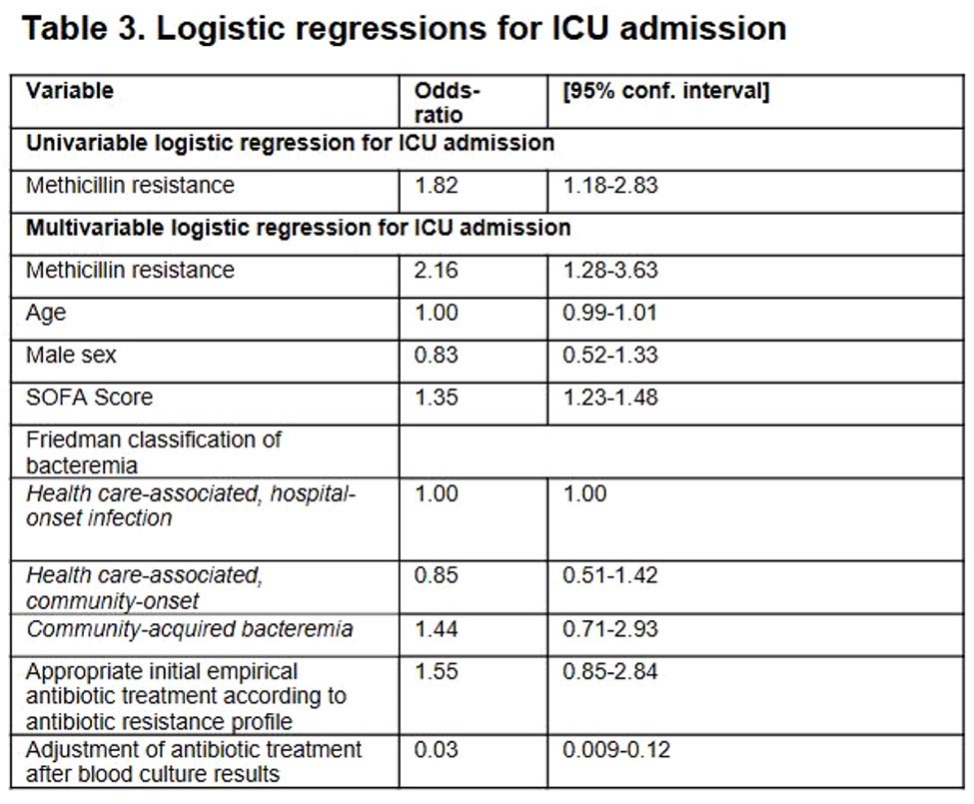
Statistical analysis: Logistic regression model adjusted for potential confounding variables. Unadjusted data present the univariable association between the exposure of interest and ICU admission. Abbreviations: OR: Odds-ratio, SOFA: Sequential Organ Failure Assessment score

**Figure:**
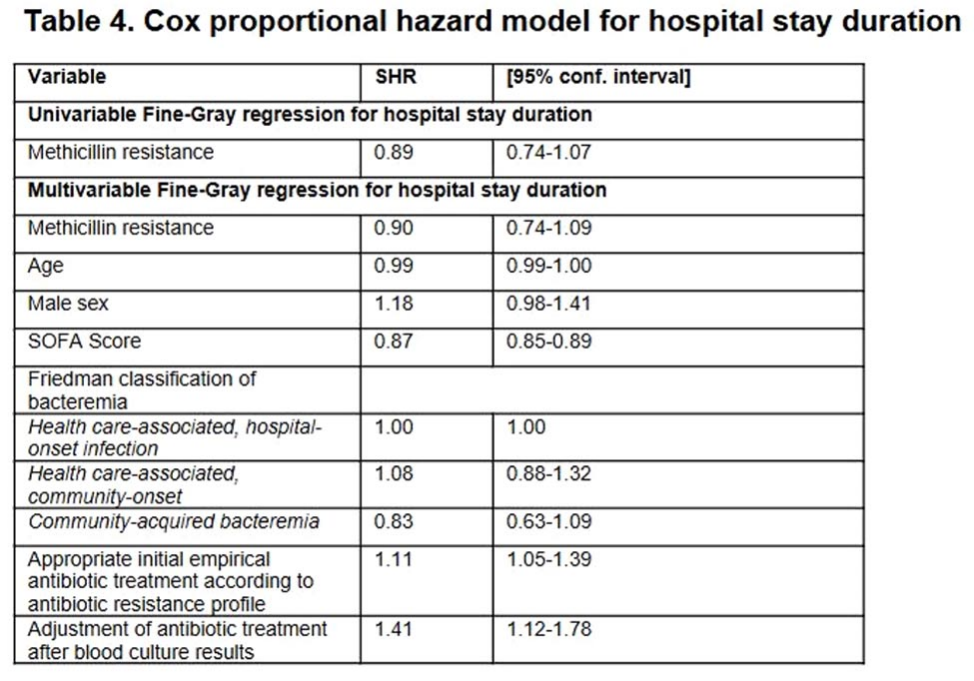
Statistical analysis: Competing risks regression (Fine and Gray) model with death as a competing risk adjusting for potential confounding variables. Unadjusted data present the univariable association between the exposure of interest and length of stay. Abbreviations: SHR: Sub-hazard ratio,, SOFA: Sequential Organ Failure Assessment score, ICU: Intensive care unit

**Results:**

Of 638 patients, 428 (67.1%) had MSSA-SAB and 210 (32.9%) had MRSA-SAB. Table 1 summarizes baseline characteristics. Multivariable analysis showed that methicillin resistance was not associated with increased in-hospital mortality; however, age, male sex, and SOFA score were independent predictors of mortality. Appropriate initial empirical antibiotic treatment and adjusted antibiotic treatment following blood culture results were independent protective factors (Table 2). Methicillin resistance was significantly associated with ICU admission (Table 3), but not with an increase length of hospital stay (Table 4).

**Conclusion:**

In our study, methicillin resistance significantly increased ICU admission rates but was not associated with higher in-hospital mortality or extended hospital stays in addition, the selection of appropriate empirical antibiotic therapy and its subsequent adjustment based on blood culture results were protective factors for hospital mortality and length of stay. These findings highlight the importance of tailored treatment strategies and early intervention in managing SAB.

**Disclosures:**

All Authors: No reported disclosures

